# Optimized process quality in certified breast centers through adherence to stringent diagnostic and therapeutic algorithms effects of structural as well as socio-demographic factors on start of therapy

**DOI:** 10.1007/s00404-022-06666-2

**Published:** 2022-08-05

**Authors:** Saskia-Laureen Herbert, Paula Hirzle, Catharina Bartmann, Tanja Schlaiß, Matthias Kiesel, Carolin Curtaz, Sanja Löb, Achim Wöckel, Joachim Diessner

**Affiliations:** Department of Obstetrics and Gynaecology, University Medical Centre Würzburg, Universitätsfrauenklinik Würzburg, Josef-Schneider-Straße 4, 97080 Würzburg, Germany

**Keywords:** Breast cancer, Delay of therapy, Prognosis, Quality of care

## Abstract

**Purpose:**

An increasing incidence of breast cancer can be observed worldwide. Since a delay of therapy can have a negative impact on prognosis, timely cancer care is an important quality indicator. By receiving treatment at a certified breast cancer center, the patient has the best chance of treatment in accordance with guidelines and the best prognosis. The identification of risk factors for a delay of therapy is of central importance and should be the basis for a continuous optimization of treatment at breast cancer centers.

**Methods:**

This retrospective study included women with breast cancer (primary diagnosis, relapse, or secondary malignancy) at the University Hospital Würzburg in 2019 and 2020. Data were retrieved from patients’ records. Correlations and regression analyses were performed to detect potential risk factors for treatment delay.

**Results:**

Patients who received the histological confirmation of breast cancer at an external institution experienced a later therapy start than those patients who received the histological confirmation at the University Hospital Würzburg itself. (35.7 vs. 32.2 days). The interval between histological confirmation and the first consultation at the University Hospital Würzburg correlated statistically significant with age, distress and distance to the hospital.

**Conclusion:**

Patients with an in-house diagnosis of breast cancer are treated more quickly than those whose diagnosis was confirmed in an external institution. We identified factors such as increased age, greater distance to the hospital as well as increased distress to prolong the time until start of oncological treatment. Intensified patient care should be offered to these subgroups.

## What does this study add to the clinical work

**Table:** 

In this study we identified risk factors for a prolonged time until start of oncological treatment. Delay of therapy can result in poorer prognosis. Thus, intensified patient care should be offered to patients with increased age, greater distance to the hospital as well as increased distress.	

## Introduction

Breast cancer is the most common malignancy in women and represents a high health burden for patients. Ferlay et al. estimate that 1.5 million women worldwide were affected in 2012 [1]. In Germany, approximately 40% of all women between the age of 35 and 59, with a new oncologic diagnosis, suffer from breast cancer [2]. In Europe, mammography screening was introduced in the 1980s [3]. Since then, there has been a considerable increase in incidence [4]. However, this varies greatly, depending on different clinical and geographical parameters [5]. The north and west of Europe show a higher incidence than the south and east [5]. Despite rising incidence rates worldwide, a decreasing mortality has been observed for several years [4]. The relative 5-year survival rate in Germany is currently about 87% [6].

With rising incidence rates, adequate care of patients must be ensured throughout the country. This can be examined and compared using quality indicators such as safety, effectiveness, efficiency, patient focus, timeliness, and appropriateness [7]. In Germany, the regularly updated guideline aims to continuously optimize the quality of care and outcome of patients. In this regard, certified breast cancer centers offer the highest probability of guideline-adherent therapy [8], which correlates with a significant improvement of the chances of cure [9].

A quality indicator in the care of breast cancer is the timely onset of oncological care [10, 11]. Delay of systemic therapy or primary surgical care is of great influence for prognosis [12–14]. Kupstas et al. demonstrated, that in breast cancer, delayed initiation of chemotherapy by 120 days after diagnosis is associated with a worse outcome [10]. Risk factors for treatment delays are multifactorial. The National Quality Measures for Breast Cancer (NQMBC) examined the time between diagnosis by biopsy and initial surgical treatment of breast cancer. A median duration of 14 working days was determined [7]. There are studies focusing on the relationship between prognosis and delay in therapy as well as the interval from diagnosis to initiation of therapy. However, the identification of risk factors for such a delay in therapy remain largely unexplored.

For this reason, the present retrospective study aims to identify factors which delayed the patients’ initial presentation to the breast cancer center or the initiation of therapy in women with newly diagnosed breast cancer (primary diagnosis/recurrence/second carcinoma). These findings can contribute to optimizing the quality of care for breast cancer patients.

## Material and methods

### Study design

The present retrospective study included patients with a diagnosis of breast cancer who were treated at the University Women's Hospital (UFK) Würzburg in 2019 and 2020. Inclusion criteria consisted of age over 18 years, female gender, adjuvant situation, and evidence of invasiveness. All breast cancer patients with tumor relapse received as a standard procedure a histological confirmation of the metastasis in order to detect the discordance of hormone- and HER2-recptors between the primary tumor and different distant metastases.

Primary diagnosis, recurrence or second carcinoma of the breast were included.

The positive confirmation of the ethics committee of the University of Würzburg is available (file no. 20200527 01).

### Instruments

Sociodemographic and clinical data were retrieved from the patient's medical record. The date of the final histological findings was utilized to determine the time of in-house diagnosis at UFK itself or at an external institution.

The interval between initial diagnosis and initiation of therapy or first presentation at the UFK was recorded in days. We selected the following parameters: date of final histological finding and first presentation at the UFK as these time points were reliably and comparably documented in all patient records.

The Charlson Comorbidity Index (CCI) was used to assess morbidity and mortality caused by additional underlying diseases. The primary disease (in this case breast cancer) is not included. Depending on the CCI, different 1-year mortality rates resulted [15].

The stress level was determined using a distress thermometer. This is a visual analog scale with values from 0 to 10 which was given to the patients during their first inpatient stay at the UFK [16].

The patients’ primary residence documented in the medical record was used to calculate the distance (in kilometers) to the UFK. By using Google maps (Google, Silicon Valley, USA), the shortest route was calculated. We could not exclude whether there was a temporary second residence for the duration of treatment.

Body mass index (BMI) was calculated as the division of body weight (in kilograms) by height (in meters) to the square.

### Statistical analysis

Statistical analysis was performed using Microsoft Excel 2019 (Microsoft, USA) and SPSS 28 (IBM, USA). The descriptive procedure includes the presentation of frequencies, means, median, and standard deviation (SD). Kolmogorov–Smirnov and Shapiro–Wilk test procedures were used to test the data for normal distribution. Mean comparison was performed using the Mann–Whitney *U* test. Pearson correlations as well as linear regression analyses were utilized to illustrate the correlations of possible factors influencing the start of therapy. Statistical significance was assumed at a *p* value of < 0.05.

## Results

### Sample description

This retrospective study included 340 UFK female patients with breast cancer. 275 (80.9%) of them presented after external histological confirmation (external group) and 65 patients (19.1%) had histological confirmation of the diagnosis at the UFK (internal group).

The mean age of the internal group was 58.06 years (SD 16.25 years) and of the external group 60.06 years (SD 13.07 years). Both groups had a mean of 1.7 children. The mean BMI was 26 for the internal group and 27 for the external group. The CCI was 1.9 for both groups, corresponding to a 1-year mortality rate of 26%. The median probability for the presence of previous psychiatric illness was 0 for both groups. The Distress Scale score, measured by using the distress thermometer, was 5.7 of 10 for the internal group and 6.2 of 10 for the external group. At the median, the patients had a tumor stage of pT1. Regarding these clinical and sociodemographic characterizations, there were no statistically significant differences between the external and internal group.

With regard to the distance from the home address to the UFK, there was a statistically significant difference (*p* < 0.01): For the internal group, the median distance was 50.1 km and for the external group it was 28.5 km (Table 1).

**Table 1:** Patient characteristics

	N		Mean		Median		SD		*p*
	int.	ext.	int.	ext.	int.	ext.	int.	ext.	
Time from diagnosis until treatment	65	275	32.23	35.7	29.00	32.00	32.00	17.10	0.026
Age	65	275	58.06	60.06	57.00	60.00	16.25	13.07	0.312
Number of children	56	257	1.70	1.74	2.00	2.00	1.09	1.08	0.692
Disease stage	65	275	1.54	1.55	1.00	1.00	0.87	0.75	0.639
BMI (kg/m2)	64	274	26.11	26.64	24.30	25.70	5.50	5.34	0.328
Charlson Comorbidity Index	65	275	1.86	1.89	2.00	2.00	1.78	1.54	0.604
Psychiatric comorbidity	65	275	0.11	0.18	0.00	0.00	0.39	0.31	0.772
Distress	51	248	5.72	6.24	5.00	6.00	2.11	2.36	0.144
Distance from home to UFK	65	275	82.77	38.40	50.10	28.50	210.71	56.42	<0.01
Time from diagnosis until 1st visit	275			16.27		15		11.17	

*int* internal, *ext* external

Statistical significance *p*<0.05

Distress measured from 0–10

### Time from diagnosis to the first visit and start of therapy

For the external group, a mean interval of 35.7 days from histological diagnosis to therapy initiation was observed. The internal group showed a significantly shorter interval of 32.2 days (*p*-value < 0.03). The median interval from external diagnosis to first presentation at the UFK is 15 days (SD 11.2).

### Factors with an impact on delayed first visit or start of therapy

Since analysis already showed a significant longer interval for the external group our main focus was identifying factors that could explain our findings. Therefore, we focused on the external group and performed further statistical analysis separately.

The following factors were examined as possible risk factors for delayed initial presentation or initiation of therapy within both groups: Age, number of children, tumor stage, primary therapy, BMI, CCI, previous psychiatric illness, distress, distance to UFK.

In the internal group there was no significant association between the named factors.

Within the external group there was a significant dependence for the parametric correlation between the interval from initial diagnosis to start of therapy and age (*p*-value 0.05; *r* = 0.12).

The same was found for the factor distress (*p*-value 0.01; *r* = 0.16). Higher age as well as higher distress both correlate with a prolonged time to start of therapy.

A significant dependence for Pearson's parametric correlation between the interval from initial diagnosis to initial presentation and age (*p*-value < 0.01; *r* = 0.19), distance (*p*-value = 0.04; *r* = 0.12) as well as CCI (*p*-value < 0.01; *r* = 0.18) could also be determined.

In the linear regression model for the time until the start of therapy with the factors age, distress and distance, a test quality of 0.037 (corrected R-squared) was received. For age and distress a significant positive effect could be found (*p*-value = 0.02 and *p*-value = 0.01). Age and distress appeared to correlate equally (beta = 0.15 and 0.17).

In the linear regression model for the time until first presentation at the UFK with the factors age and distance, a test quality of 0.055 (corrected R-squared) was analyzed. A significant positive effect was found for age and distance (*p*-value < 0.01 and *p* = 0.02). Both factors proved to be almost equal (beta = 0.20 and 0.14).

## Discussion

The incidence of breast cancer is increasing worldwide. However, there are significant differences in age and geography [17]. For the quality of patient care, a growing incidence represents a challenge for the healthcare system.

In order to maintain the quality of care and outcome of affected patients at a high level and to further improve it, the treatment of breast cancer in Germany is carried out according to regularly updated guidelines. Studies have shown that guideline-adherent therapy, which is based on constantly improving scientific progress, significantly improves the chances of cure [9]. Scientific studies have been able to prove that treatment in certified breast cancer centers offers the best chance of guideline-compliant treatment and survival [8, 18, 19]. In Germany, approximately 80% of breast cancer patients are treated at one of more than 270 breast cancer centers distributed nationwide. A comprehensive set of regulations coordinates the personnel as well as structural and qualitative conditions of a breast cancer center, which are verified by regular, independent inspections [8, 18].

In order to also examine the influence of the affiliation of affected breast cancer patients with a certified breast cancer center, we investigated the effects of sociodemographic and structural factors on the initiation of therapy. This showed that patients, who received histological confirmation of the diagnosis outside of our certified breast center, experienced a significantly later onset of therapy. The median time between histological diagnosis and first presentation at our breast center was 15 days. For this interval, both, the age of the patient and the distance to the UFK statistical showed statistically significant influence. The older the patient and the further the distance, the greater the time interval. Regarding the time interval between histologic diagnosis and therapy initiation, there is a significant correlation with the patient's age and distress at the time of hospital admission. The older the patient and the higher the stress level, the longer the time interval.

The “National Quality Measures for Breast Cancer” study describes that initial surgical care was found to take 14 working days between needle biopsy and initial cancer surgery. According to this study, it took this time-period to obtain needle biopsy results, surgical consultation and surgery scheduling [7]. In our analysis we observed, however, a mean interval of 35.7 days from histological diagnosis to therapy initiation for the external group and 32.2 days for the internal group.

The considerable time difference of 21 respectively 18 days is multifactorial. First of all, Kaufman et al. calculated with working days whereas we included Saturday and Sunday for the time calculation [7]. Another important factor represents the time-period of the analysis. For our study we refer to the years 2019 and 2020, Kaufman et al. analysed a time interval between 2005 and 2007 [7]. During the last 15 respectively 17 years therapy algorithm of breast cancer as well as diagnostic options have increased and individualized considerably. In particular, cross-sectional imaging as computed tomography (CT) and magnetic resonance imaging (MRI) have been refined to an extent over the past 15 years that the diagnostic process is lengthened. Moreover, the application of multigene assays like Oncotype DX® and neoadjuvant therapy strategies extend the time interval for the development of a customized, individualized therapy concept at the beginning of each treatment [7].

The UFK Würzburg is one of the 270 certified breast centers in Germany. An important criterion for these centers is the interdisciplinarity of experts in diagnostics, surgery, radiation and drug therapy. The treatment goal of this team was the development of a customized, individualized therapy concept at the beginning of each treatment. This results in a high quality of structure, process and outcome with close networking of the various departments involved in the therapy process. By using patient managers and standardized, stringent diagnoses and therapy plans, internal processes in particular can be implemented in an optimized manner. Consequently, time loss can be reduced to a minimum. As described above, there still is a need for further optimization concerning externally diagnosed patients. Expanding the appointment capacity and providing support in terms of mobility are possible options for improvement. Despite the pandemic, appointment scheduling for oncology patients at the UFK remained unchanged.

A delay in therapy can be divided according to its causes. It can derive from either the patient herself, or by the healthcare provider. For our study, the period between histological findings and therapy initiation was of particular interest, as this interval may reflect the quality of care. In addition, this period can be accurately timed by the presence of the date of the findings and the start of therapy.

Concerning the question of the significance of the delay in therapy on the further prognosis of breast cancer, Bleicher et al. were able to define periods within which a deterioration of the clinical outcome is not yet to be expected [20]. The interval between diagnosis and surgery should be less than 90 days and the interval between diagnosis and chemotherapy should be less than 120 days. A delay of more than three months is associated with a worse prognosis [20–22]. Primary therapy initiation within the recommended time frame is shown for both groups in our study. Delays should also be avoided from a health economic perspective, as advanced disease is considered more costly in terms of care [23].

The incidence of breast cancer increases with age. While the estimated risk of developing breast cancer is 1/43 between the ages of 50 to 59 years, the risk increases to 1/15 at ages > 70 years [24]. The median age of onset for breast cancer is approximately 64 years [6]. About 50% of patients are older than 65 years at diagnosis [25]. This age group accounts for more or less 60% of all breast cancer-associated deaths [24, 26]. 20% of the population is estimated to be older than 65 years by 2030 [27]. Increasing comorbidities are also recorded with advanced age [28, 29]. Thus, the expansion of this age group is also medically significant. While triple negative breast cancer is the most common subgroup in the age of 50 to 59 years, luminal A tumors are mainly found in the age group of 60 to 69 and over 70 years [30]. The time interval between symptom perception and physician consultation is often delayed in the patient group over 65 years of age [31, 32]. Hence, despite the less aggressive subtype, initial presentation is more frequent with already advanced tumor disease [33]. Exfoliated breast cancer presents significantly more frequently in the over-75 years age group than in younger patients [34]. In this situation, the time factor becomes even more important with regard to the nursing care aspect and the assessment of operability. Therefore, it seems even more important to support this group of patients with a breast center based care as soon as possible. Reasons for a delayed consultation of older patients can be social and organizational. While younger patients can usually visit the breast center on their own, older patients often lack the necessary mobility. The median distance for the external group is only half of the distance of the internal group. The further the distance of the external group, the more delayed the first presentation. This correlation is also described in the literature. Dickens et al. were able to identify two factors negatively influencing the time to first presentation: a radius of more than 20 km to the health care center as well as an increased age [35]. From this point of view, a certain significance can be attributed to the factor of mobility. The support of elderly patients with specialized transport service could easily be improved. Patients could also be offered professional appointment coordination for prompt presentation at a breast center. Finally, however, it must be noted that little literature is available on the topic of care/connection of older patients and thus further studies are necessary.

Despite the urgency of oncological treatment, it must also be taken into account, that with increasing age, the extent of comorbidities also increases [36]. First, these comorbidities themselves may have an impact on the prognosis of the patient [15]. Second, this may also lead to a limitation of therapeutic options. Since comorbidities are independent risk factors for survival, it remains unclear to what extent mortality may be influenced.

Another risk factor we identified is the patient's stress level as measured by the distress thermometer. Based on a defined cut-off value of 5 of 10 in the distress thermometer, a sensitivity of 84% and a specificity of 47% is shown for detecting anxiety and/ or depression [37]. The median cut-off was reached in both the internal and the external group. Jassem et al. showed that anxiety is a risk factor for treatment delay [23]. Nevertheless, other studies could attribute less importance to the anxiety factor [31, 38]. However, because the stress level in our study was collected at the time of the first inpatient stay, it remains unclear how high the stress was when the histologic diagnosis was received. This fact represents a limitation of this study.

Overall, it must be noted that the adjusted R2 for all factors identified must be considered rather low. This may be due to the risk factors themselves basing on a multifactorial relationship (Tables 2, 3 and 4).

**Table 2:** Regression model results (Adjusted R-squared = .037) with regression coefficients B indicating 95% confidence interval (95% CI), standardized regression coefficients beta and *p* value *p*

	B [95%-CI]	Beta	*p*
Constant	17,89 (6,57; 29,22)		0.002
Age	0,18 (0,03; 0,33)	0.15	0.023
Distress	1,14 (0,30; 1,98)	0.17	0.008
Distance	0,02 (− 0,01; 0,05)	0.07	0.253
R^2	0.049		
Adjusted R^2	0.037

**Table 3:** Regression model results (Adjusted R-squared = .055) with regression coefficients B indicating 95% confidence interval (95% CI), standardized regression coefficients beta and *p*-value *p*

	B [95%-CI]	Beta	*p*
Constant	4,88 ( – 1,37; 11,12)		0.126
Age	0,17 (0,07; 0,27)	0.20	< 0.001
Distance	0,03 (0,01; 0,05)	0.14	0.019
R^2	0.235		
Adjusted R^2	0.055

**Table 4:** Pearson´s correlation

		Age	Children	Tumor stage	Primary therapy: surgery - chemotherapy - radiatio - endocrine	Time from diagnosis until treatment	BMI	Charlson comorbidity index	Psychiatric disorder	Distress	Distance to UKW	Reason for diagnostic: symptoms - screening - aftercare - by chance	Time from diagnosis until first visit (ext.)
Time from diagnosis until treatment (int.)	Correlation coefficient r	– 0.13	0.14	– 0.17	– 0.10	1.00	– 0.13	– 0.08	0.16	– 0.11	– 0.15	0.12	
	Significance	0.31	0.33	0.17	0.41		0.32	0.55	0.20	0.44	0.24	0.38	
	*N*	63.00	54.00	63.00	63.00	63.00	62.00	63.00	63.00	49.00	63.00	58.00	
Time from diagnosis until treatment (ext.)	Correlation coefficient r	,12^*^	0.07	0.12	– 0.06	1.00	– 0.02	0.11	0.01	,16^*^	0.08	0.12	
	Significance	0.05	0.30	0.05	0.29		0.72	0.08	0.87	0.01	0.16	0.07	
	*N*	275.00	257.00	275.00	275.00	275.00	274.00	275.00	275.00	248.00	275.00	246.00	
Time from diagnosis until first visit (ext.)	Correlation coefficient r	,19^**^	0.01	0.06	-0.11	0.10	0.07	,18^**^	0.00	0.06	,12^*^	,23^**^	1.00
	Significance	0.00	0.82	0.34	0.08	0.10	0.27	0.00	0.96	0.35	0.04	0.00	
	*N*	275.00	257.00	275.00	275.00	275.00	274.00	275.00	275.00	248.00	275.00	246.00	275.00

*int* internal, *ext* external

**Correlation is significant at the 0.01 level (2-sided)

*Correlation is significant at the 0.05 level (2-sided)

Another major limitation of this analysis is the monocentric character and the focus on the actual medical care situation in Germany. Current realities in the healthcare field vary heavily, depending on the national health care system. It would be very informative to expand this analysis to an international level.

In this retrospective analysis, we were able to show that older patients with a greater local distance to the oncology center, as well as patients with a high psychological burden, are affected by delays in the course of therapy.

With regard to the quality of care, the time interval from diagnosis to the start of therapy plays an important role, especially for these groups of patients. In order to further optimize the quality of care for breast cancer patients, special attention should be paid to these identified patient groups. The affiliation with certified breast cancer centers appears to be a promising step to optimize the stringency of the therapy algorithm.

## Data Availability

The datasets generated during and/or analysed during the current study are available from the corresponding author on reasonable request.

## References

[1] Ferlay J, Soerjomataram I, Dikshit R, Eser S, Mathers C, Rebelo M, Parkin DM, Forman D, Bray F (2015). Cancer incidence and mortality worldwide: sources, methods and major patterns in GLOBOCAN 2012. Int J Cancer.

[2] Bundesamt S (2021) Gesundheitsberichterstattung des Bundes. https://www.gbe-bund.de/gbe/abrechnung.prc_abr_test_logon?p_uid=gast&p_aid=0&p_knoten=FID&p_sprache=D&p_suchstring=9508.

[3] Botha JL, Bray F, Sankila R, Parkin DM (2003). Breast cancer incidence and mortality trends in 16 European countries. Eur J Cancer.

[4] Katalinic A, Eisemann N, Kraywinkel K, Noftz MR, Hubner J (2020). Breast cancer incidence and mortality before and after implementation of the German mammography screening program. Int J Cancer.

[5] Ferlay J, Parkin DM, Steliarova-Foucher E (2010). Estimates of cancer incidence and mortality in Europe in 2008. Eur J Cancer.

[6] Robert-Koch-Institut (2021) Krebsregisterdaten Mammakarzinom. https://www.krebsdaten.de/Krebs/DE/Content/Krebsarten/Brustkrebs/brustkrebs_node.html.

[7] Kaufman CS, Shockney L, Rabinowitz B, Coleman C, Beard C, Landercasper J, Askew JB, Wiggins D, Quality Initiative C (2010). National Quality Measures for Breast Centers (NQMBC): A Robust Quality Tool. Ann Surg Oncol.

[8] Kowalski C, Ferencz J, Brucker SY, Kreienberg R, Wesselmann S (2015). Quality of care in breast cancer centers: results of benchmarking by the German Cancer Society and German Society for Breast Diseases. The Breast.

[9] Wöckel A, Varga D, Kurzeder C, Atassi Z, Wolters R, Wischnewsky M, Kreienberg R (2010). Guideline adherence in primary breast cancer. Senologie.

[10] Kupstas AR, Hoskin TL, Day CN, Habermann EB, Boughey JC (2019). Effect of surgery type on time to adjuvant chemotherapy and impact of delay on breast cancer survival: a national cancer database analysis. Ann Surg Oncol.

[11] Rosselli Del Turco M, Ponti A, Bick U, Biganzoli L, Cserni G, Cutuli B, Decker T, Dietel M, Gentilini O, Kuehn T, Mano MP, Mantellini P, Marotti L, Poortmans P, Rank F, Roe H, Scaffidi E, van der Hage JA, Viale G, Wells C, Welnicka-Jaskiewicz M, Wengstöm Y, Cataliotti L (2010). Quality indicators in breast cancer care. Eur J Cancer.

[12] Lohrisch C, Paltiel C, Gelmon K, Speers C, Taylor S, Barnett J, Olivotto IA (2006). Impact on survival of time from definitive surgery to initiation of adjuvant chemotherapy for early-stage breast cancer. J Clin Oncol.

[13] Hershman DL, Wang X, McBride R, Jacobson JS, Grann VR, Neugut AI (2006). Delay of adjuvant chemotherapy initiation following breast cancer surgery among elderly women. Breast Cancer Res Treat.

[14] de Melo GD, Lei X, Giordano SH, Valero V, Barcenas CH, Hortobagyi GN, Chavez-MacGregor M (2020). Impact of Delayed Neoadjuvant Systemic Chemotherapy on Overall Survival Among Patients with Breast Cancer. Oncologist.

[15] Land LH, Dalton SO, Jensen MB, Ewertz M (2012). Influence of comorbidity on the effect of adjuvant treatment and age in patients with early-stage breast cancer. Br J Cancer.

[16] Mehnert A, Müller D, Lehmann C, Koch U (2006). Die deutsche version des NCCN distress-thermometers: empirische Prüfung eines screening-instruments zur erfassung psychosozialer belastung bei krebspatienten. Z Psychiatr Psychol Psychother.

[17] Bray F, McCarron P, Parkin DM (2004). The changing global patterns of female breast cancer incidence and mortality. Breast Cancer Res.

[18] Beckmann MW, Brucker C, Hanf V, Rauh C, Bani MR, Knob S, Petsch S, Schick S, Fasching PA, Hartmann A (2011). Quality assured health care in certified breast centers and improvement of the prognosis of breast cancer patients. Oncology Research and Treatment.

[19] Kemeny MM (2017) The effect of centralizing breast cancer care in an urban public hospital. Queens Cancer Center, NYC Health + Hospitals/Queens, Icahn School of Medicine at Mount Sinai, New York

[20] Bleicher RJ (2018). Timing and delays in breast cancer evaluation and treatment. Ann Surg Oncol.

[21] Richards M, Westcombe A, Love S, Littlejohns P, Ramirez A (1999). Influence of delay on survival in patients with breast cancer: a systematic review. The Lancet.

[22] Afzelius P, Zedeler K, Sommer H, Mouridsen HT, Blichert-Toft M (1994). Patient's and doctor's delay in primary breast cancer: Prognostic implications. Acta Oncol.

[23] Jassem J, Ozmen V, Bacanu F, Drobniene M, Eglitis J, Lakshmaiah KC, Kahan Z, Mardiak J, Pieńkowski T, Semiglazova T (2014). Delays in diagnosis and treatment of breast cancer: a multinational analysis. Eur J Public Health.

[24] Siegel R, Ma J, Zou Z, Jemal A (2014). Cancer statistics. CA Cancer J Clin.

[25] Crivellari D, Aapro M, Leonard R, Gv M, Brain E, Goldhirsch A, Veronesi A, Muss H (2007). Breast cancer in the elderly. J Clin Oncol.

[26] DeSantis C, Siegel R, Bandi P, Jemal A (2011). Breast cancer statistics. CA Cancer J Clin.

[27] Yancik R (1997). Cancer burden in the aged: an epidemiologic and demographic overview. Cancer.

[28] Yancik R, Havlik RJ, Wesley MN, Ries L, Long S, Rossi WK, Edwards BK (1996). Cancer and comorbidity in older patients: a descriptive profile. Ann Epidemiol.

[29] Coebergh J, Janssen-Heijnen M, Post P, Razenberg P (1999). Serious co-morbidity among unselected cancer patients newly diagnosed in the southeastern part of The Netherlands in 1993–1996. J Clin Epidemiol.

[30] McGuire A, Brown JAL, Malone C, McLaughlin R, Kerin MJ (2015). Effects of Age on the Detection and Management of Breast Cancer. Cancers.

[31] Arndt V, Stürmer T, Stegmaier C, Ziegler H, Dhom G, Brenner H (2002). Patient delay and stage of diagnosis among breast cancer patients in Germany–a population based study. Br J Cancer.

[32] Facione NC, Miaskowski C, Dodd MJ, Paul SM (2002). The self-reported likelihood of patient delay in breast cancer: new thoughts for early detection. Prev Med.

[33] Barthélémy P, Heitz D, Mathelin C, Polesi H, Asmane I, Litique V, Rob L, Bergerat JP, Kurtz JE (2011). Adjuvant chemotherapy in elderly patients with early breast cancer. Impact of age and comprehensive geriatric assessment on tumor board proposals. Crit Rev Oncol Hematol.

[34] Sanguinetti A, Ragusa M, De Falco M, Sperlongano P, Calzolari F, Parmeggiani D, Misso C, Piatto A, Parmeggiani U, Avenia N (2007). Locally advanced breast cancer in elderly patients: treatment standardised or tailored to individual needs?. Chir Ital.

[35] Dickens C, Joffe M, Jacobson J, Venter F, Schüz J, Cubasch H, McCormack V (2014). Stage at breast cancer diagnosis and distance from diagnostic hospital in a periurban setting: a South African public hospital case series of over 1,000 women. Int J Cancer.

[36] Charlson M, Szatrowski TP, Peterson J, Gold J (1994). Validation of a combined comorbidity index. J Clin Epidemiol.

[37] Mehnert A, Mueller D, Lehmann C, Koch U (2006). The German version of the NCCN distress thermometer: validation of a screening instrument for assessment of psychosocial distress in cancer patients. Zeitschrift fur Psychiatrie Psychologie Und Psychotherapie.

[38] Cobb B, Clark RL, McGuire C, Howe C (1954). Patient-responsible delay of treatment in cancer, a social psychological study. Cancer.

